# Diversity of Variable Number Tandem Repeat Loci in Shigella Species Isolated from Pediatric Patients

**Published:** 2015

**Authors:** Reza Ranjbar, Mojtaba Memariani, Hamed Memariani

**Affiliations:** 1 *Molecular Biology Research Center, Baqiyatallah University of Medical Sciences, Tehran, Iran.*; 2 *Department of Molecular and Cell Biology, Faculty of Basic Sciences, University of Mazandaran, Babolsar, Iran.*; 3 *Biotechnology Research Center, Pasteur Institute of Iran, Tehran, Iran.*

**Keywords:** *Shigella*, MLVA, genotyping

## Abstract

Multilocus variable number tandem repeat (VNTR) analysis (MLVA) is a new typing method with several advantages compared to other methods. Dissemination of *Shigella* is highly significant in developing countries. Whilst *Shigella* is becoming increasingly important as an etiologic agent of pediatric shigellosis in Iran, little is known about the genetic diversity of the local strains. Therefore, the aim of this study was to describe the genetic diversity of *Shigella* species isolated from pediatric patients in Tehran, Iran. A total of 53 *Shigella* isolates were obtained from 1070 patients with diarrhea (less than 12 years of age). All isolates were identified by routine biochemical and serological tests. The confirmed *Shigella* isolates were further serogrouped (by the slide agglutination) using slide agglutination method. MLVA assay with the seven loci resolved 53 *Shigella* isolates into 36 different genotypes. Almost all the isolates were classified into five clonal complexes. Furthermore, our MLVA assay could effectively distinguish the four *Shigella *species. This study has provided valuable insights into the genetic heterogeneity of *Shigella* species in Tehran, Iran. Our findings can be helpful for further epidemiological surveillance of *Shigella *species in this country in the future.

Diarrheal diseases are one of the most important causes of children mortality, accounting for around two million deaths annually. Bacillary dysentery, also known as shigellosis, causes a significant proportion of morbidity and mortality, especially in children with diarrhea in developing countries (1). Shigellosis is primarily a disease of poor, crowded communities which do not have adequate sanitation or clean water. The genus *Shigella* includes four species: *S. dysenteriae, S. flexneri*, *S. boydii*, and *S. sonnei*. Of these species, shigellosis is predominantly caused by *S. flexneri* in developing countries especially in Asia, whereas *S. sonnei* is predominant in industrialized countries (2, 3).

Genotyping methods provide useful informa-tion for establishing the genetic relatedness among isolates for the purposes of epidemiological investigation and evolutionary studies. A variety of molecular typing methods have been developed for *Shigella* species, including pulsed- field gel electrophoresis (PFGE) (4-6), multilocus sequence typing (MLST) (7), multilocus variable-number tandem-repeat (VNTR) analysis (MLVA) (8-11), ribotyping (12-15), plasmid profiling (4, 16) and repetitive sequence-based PCR (Rep-PCR) (7, 17). Although PFGE is still a golden standard for genotyping and source tracking of food-borne pathogens, it is laborious, expensive, often difficult to interpret, and requires rigorous standardization. Furthermore, it needs experienced personnel in order to achieve reliable, consistent, and reproducible results. By contrast, MLVA is a PCR-based genotyping method which is rapid, relatively cheap and easy to perform. This method targets multiple VNTR loci and relies on the detection of different copy numbers inside each locus (18).

Dissemination of *Shigella* is highly significant in developing countries. Whilst *Shigella* is becoming increasingly important as an etiologic agent of pediatric shigellosis in Iran (2, 3, 19), little is known about the genetic diversity of the local strains. To the best of our knowledge, this is the first study on genotype of *Shigella* by MLVA in Iran. Therefore, the aim of this study was to describe the genetic diversity of *Shigella* species isolated from pediatric patients in Tehran, Iran.

## Materials and methods

**Bacterial strains**

In this study, from June 2008 to October 2010, a total of 53 *Shigella* isolates were obtained from 1070 patients with diarrhea (less than 12 years of age) in Tehran, Iran. All of the isolates were identified by routine biochemical and serological tests. The confirmed *Shigella* isolates were further serogrouped by the slide agglutination test (Mast Diagnostic, Merseyside, UK). The verified isolates were preserved at -70°C in tripticase soy broth (TSB) with 25% (v/v) glycerol for further analysis (3). All ethical issues were considered. Life, health, dignity, integrity, right to self-determination, privacy, and confidentiality of personal information of research subjects were protected in this study.

**DNA extraction**

Each* Shigella *isolate was plated on nutrient agar and incubated overnight at 37 °C. A single colony was removed from the plate, suspended in 200 μl of sterile deionized water and boiled for 15 min. After centrifugation at 8,000x g for 6 min, the supernatant was transferred into a new tube for subsequent PCR analysis.

**MLVA assay**

The VNTR loci selected along with the primers used for MLVA genotyping were those of Gorge et al., which were seven loci; ms06, ms07, ms09, ms11, ms21, ms23, and ms32 (20). The list of primers used are shown in Table 1. For each loci, PCR was performed in 25 μl volume containing 1X PCR buffer (50 mmol/L KCL, 10 mmol/L Tris, pH 9), 2.5 mmol/L MgCl_2_, 0.2 mmol/L of each primer with 0.5 U TaqDNA polymerase (CinnaGen Co., Iran), and 3 μl of crude DNA extract. Cycling conditions for all PCR reactions were 93 °C for 5 min, followed by 34 cycles of 93 °C for 30 s, 55 °C for 1 min, and 72 °C for 1 min. A final extension of 72 °C for 6 min was also employed. The PCR products were run on 1.5% agarose gels, stained with ethidium bromide, and visualized under UV transillumination.

**Data analysis**

The repeat units of each locus were determined by subtracting the size of flanking regions (offset size) from PCR amplicon size and then dividing the difference by the repeat size. The result was then rounded to the nearest integer value to simplify reporting of MLVA data. Repeat units were imported into Microsoft Excel. The minimum spanning tree (MST) was constructed with a categorical coefficient based on allelic profiles of the *Shigella* strains using the trial version of Ridom MLVA compare software (Ridom® GmbH, Germany). MST is a convenient complementary tool to cluster multiple isolates and visualize the relative diversity within different lineage. A dendrogram of genetic relationships was also generated using the unweighted pair group method with arithmetic averages (UPGMA) method (21). *S.flexneri* Sf2457T (serotype 2a) was also included as an outgroup.

## Results

**Fig. 1:**
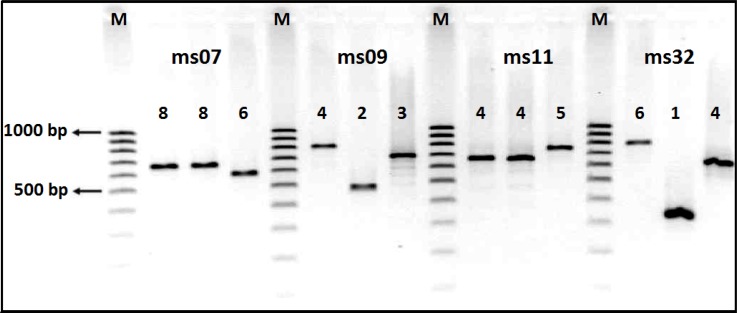
Polymorphism of ms07, ms09, ms11, and ms32 loci in different *Shigella *species. The number of repeats can be directly deduced by manual reading. The numbers above the PCR amplicons provide the repeat numbers of each VNTR locus. Lanes M, DNA markers

**Fig. 2:**
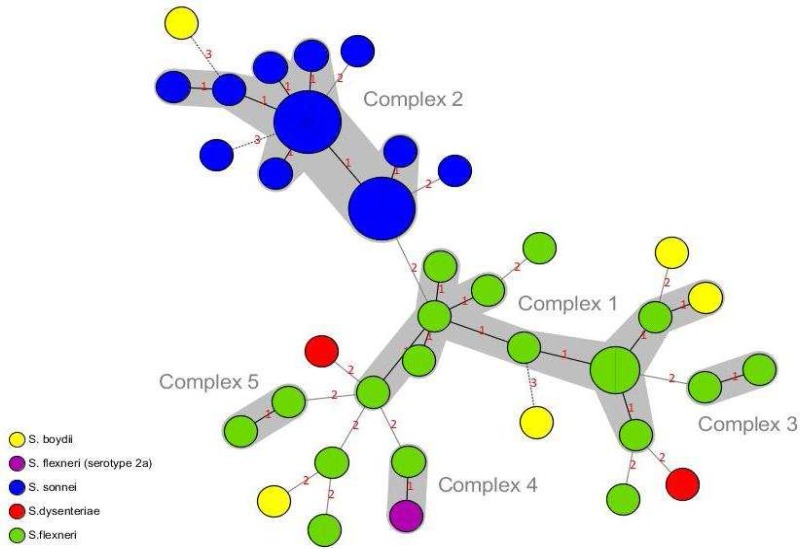
Minimum spanning tree (MST) for *Shigella* isolates. Each circle represents one strain with a unique genotype (MLVA profile). The size of the circles indicates the number of isolates. The number of loci which differ between two MLVA types is indicated on the lines connecting the MLVA types. Clonal Complexes (CCs) were indicated by grey halos. The color of the circles corresponds to *Shigella* species. *S. flexneri* Sf2457T [serotype 2a] was also used as an outgroup

**Fig. 3:**
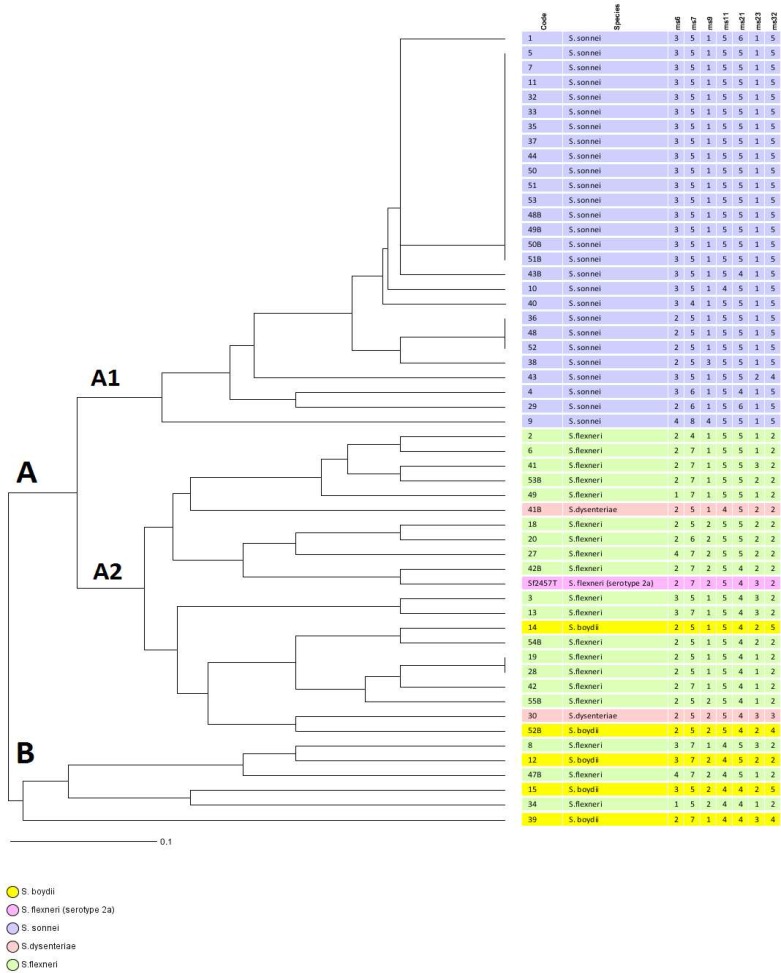
UPGMA analysis of *Shigella* isolates based on VNTR profiles. The highlighted colors represent *Shigella* species. *S. flexneri* Sf2457T [serotype 2a] was also used as an outgroup

Of the 53 *Shigella* isolates, *S. sonnei *(50.9%, n= 27) was the most common, followed by *S. flexneri *(35.8%, n= 19), *S. boydii *(9.4%, n= 5), and *S. dysenteriae* (3.8%, n= 2). The age distribution data revealed that *Shigella* was isolated from 40 (75.5%) cases in the 2-5 year-old, 11 (20.7%) in the 6-12 year-old and 2 (3.8%) in ≤ one year-old age groups. Furthermore, this difference was statistically significant (P< 0.001), suggesting that the peak age of shigellosis was 2-5 years in children. MLVA based on seven VNTR loci was carried out to characterize the *Shigella* isolates (Figure 1). Overall, *Shigella* isolates were discriminated into 36 different MLVA types (genotypes). Almost all of the strains were classified into five clonal complexes (CCs). CCs are defined as a group of allelic profiles in which every profile shares at least 5 loci in common with at least an other member of the group. MST analysis showed that most of the *S. sonnei* strains fell into one CC (i.e. CC2), whereas most of the *S. flexneri* strains were categorized into three CCs (i.e. CC1, CC3, and CC5) (Figure 2). The UPGMA dendrogram with detail information including isolate code, species, and allelic profile is shown in Figure 3. All the *Shigella* isolates were categorized into two main clusters (i.e. cluster A and cluster B). Cluster A consisted of two subclusters, which represented most of our isolates (n=47). Interestingly, subcluster A1 contains only *S. sonnei* isolates, whereas subcluster A2 is relatively diverse. Cluster B only includes some strains of *S. boydii* and *S. flexneri*.

## Discussion

In developing countries, poor hygiene, lack of clean water supply and overcrowded living conditions facilitate *Shigella* transmission, mainly through direct oral-fecal contamination. While *S .flexneri* is primarily endemic in developing world*, S.sonnei* is more commonly reported in industri-alized countries. Based on a surveillance study in six countries across Asia, *S. flexneri* was the most frequently isolated *Shigella* species in Bangladesh, China, Indonesia, Pakistan, and Vietnam, while *S. sonnei* was the predominant species in Thailand.

**Table 1:** PCR primers for specific VNTR loci and their corresponding tandem repeat (TR) and offset sizes in reference strain *S. flexneri *2457T chromosome

**VNTR locus**	**Primer sequence (5' to 3')**	**Tandem Repeat** **(TR) size, bp**	**Offset size, bp**	**References**
ms06	F: AAA CGG GAG AGC CGG TTA TT	39	223	20
	R: TGT TGG TAC AAC GGC TCC TG			
ms07(CVN001)	F: GTC AGT TCG CCC AGA CAC AG	39	353	20
	R: CGG TGT CAG CAA ATC CAG AG			
ms09	F: GTG CCA TCG GGC AAA ATT AG	179	177	20
	R: CCG ATA AGG GAG CAG GCT AGT			
ms11	F: GAA ACA GGC CCA GGC TAC AC	96	286	20
	R: CTG GCG CTG GTT ATG GGT AT			
ms21	F: GCT GAT GGC GAA GGA GAA GA	141	82	20
	R: GGG AGT ATG CGG TCA AAA GC			
ms23	F: GCT CCG CTG ATT GAC TCC TT	375	209	20
	R: CGG TTG CTC GAC CAC TAA CA			
ms32	F: GAG ATT GCC GAA GTG TTG C	101	254	20
	R: AAC TGG CGG CGT TTA TCA AG			

However, *S. boydii* is mainly restricted to the Indian subcontinent, being the second most abundant species isolated in Bangladesh (22). Similar to many previous studies from developing countries (22-24), *S. flexneri* is still predominant species in many parts of Iran (25, 26). However, in the present study, almost half of the isolates belonged to *S. sonnei* which may suggest the possible succession of *S. flexneri *by *S. sonnei *in urban areas in Iran as the standard of living improved, as inferred from observations obtained from industrialized countries (2, 3).

MLVA is an effective molecular typing technique based on counting the number of VNTR repeats and has advantages such as rapidity, ease, and convenience of interpretation. Since VNTR loci have various degrees of variability, they are useful molecular markers for phylogenetic and evolutio-nary study of bacterial pathogens. MLVA has been applied to investigate the clonal relationships among isolates of *E. coli* and* Shigella* species (20). In this study, we used MLVA profiles to establish phylogenetic relationships among 53 *Shigella *isolates. Moreover, our MLVA assay could effectively distinguish the four *Shigella *species. In the current survey, *S. flexneri* isolates showed higher diversity compared to *S. sonnei* isolates. This is not a surprise because unlike *S.sonnei, *which has only one serotype, *S. flexneri *has eight serotypes with at least 12 subserotypes (27). In recent years, several MLVA assays have been developed for *Shigella* species. In a study conducted by Qu et al. (2012) in Beijing, China, 180 strains of *S. flexneri* and *S. sonnei* were divided into 84 MLVA subtypes based on 10 VNTR loci (28). In a study by Koh et al. 40 strains of *S. sonnei* strains were isolated during the years 1997–2000, and during 2007–2009 they were classified into different clones based on 7 VNTR loci. They demonstrated that *S. sonnei* isolates with no epidemiological linkage were clustered together which could be due to traveling within the country and/or person-to-person spread of a particular strain over a long period of time with minor genetic changes (8). Izumiya et al. analyzed *S. sonnei* strains isolated from cases associated with foreign travel in Japan by MLVA and they showed that these isolates could be grouped into several clusters based on their countries of origin (11). In another study, Chiou et al. used 26 VNTR loci for phylogenetic analysis of 916 *S. sonnei* isolates collected in Taiwan over 9 years (10). Wang et al. also developed and evaluated MLVA assay based on 36 VNTR loci for discriminating the different serotypes of *S. flexneri *(27). Generally, these studies showed that MLVA has the potential to replace other genotyping methods as a standard method for *Shigella* typing. However, lack of standardization of the methodology and interpretive criteria is problematic and hinders comparison of data between laboratories (9).

On the other hand, it is important to note that most of these mentioned MLVA schemes require a high precision of DNA length measurement, for instance, by microcapillary electrophoresis and fluorescent markers because the selected VNTRs have very short repeat units. Moreover, developing countries have limited accessibility to such equipment and their expenditures would be a major obstacle for many laboratories (20, 29). In this study, we exploited VNTR markers which can be easily analyzed by eye on agarose gels. Hence, this assay can be performed in a laboratory equipped with simple equipment.

The present study provided valuable insights into the genetic heterogeneity of *Shigella* species in Tehran. We hope that our findings can be helpful for further epidemiological surveillance of *Shigella *species in our country in the future. Finally, this study showed that MLVA is a promising typing technique for epidemiological studies of *Shigella* species due to low cost, the speed of analysis, and the ability to produce numerical data which can be easily shared among laboratories.
